# COVID-19 Preventive Practices, Psychological Distress, and Reported Barriers to Healthcare Access during the Pandemic among Adult Community Members in Sub-Saharan Africa: A Phone Survey

**DOI:** 10.4269/ajtmh.22-0349

**Published:** 2022-12-12

**Authors:** Nega Assefa, Yasir Y. Abdullahi, Elena C. Hemler, Bruno Lankoande, Isabel Madzorera, Dongqing Wang, Abbas Ismail, Angela Chukwu, Firehiwot Workneh, Frank Mapendo, Ourohiré Millogo, Sulemana Watara Abubakari, Lawrence Gyabaa Febir, Isaac Lyatuu, Kassoum Dianou, Till Baernighausen, Abdramane Soura, Kwaku Poku Asante, Emily Smith, Said Vuai, Alemayehu Worku, Japhet Killewo, Mary Mwanyika-Sando, Yemane Berhane, Ali Sie, Raji Tajudeen, Ayo Oduola, Wafaie W. Fawzi

**Affiliations:** ^1^ College of Health and Medical Sciences, Haramaya University, Harar, Ethiopia;; ^2^ Department of Global Health and Population, Harvard T.H. Chan School of Public Health, Harvard University, Boston, Massachusetts;; ^3^ Institut Supérieur des Sciences de la Population, University of Ouagadougou, Ouagadougou, Burkina Faso;; ^4^ College of Natural and Mathematical Sciences, University of Dodoma, Dodoma, Tanzania;; ^5^ Department of Statistics, University of Ibadan, Ibadan, Nigeria;; ^6^ Addis Continental Institute of Public Health, Addis Ababa, Ethiopia;; ^7^ Africa Academy for Public Health, Dar es Salaam, Tanzania;; ^8^ Nouna Health Research Center, Nouna, Burkina Faso;; ^9^ Kintampo Health Research Centre, Research and Development Division, Ghana Health Service, Kintampo North Municipality, Bono East Region, Ghana;; ^10^ Heidelberg Institute of Global Health, University of Heidelberg, Heidelberg, Germany;; ^11^ Africa Health Research Institute, KwaZulu-Natal, South Africa;; ^12^ Department of Global Health, Milken Institute School of Public Health, George Washington University, Washington, District of Columbia;; ^13^ Department of Exercise and Nutrition Sciences, Milken Institute School of Public Health, George Washington University, Washington, District of Columbia;; ^14^ Department of Preventive Medicine, School of Public Health, College of Health Sciences, Addis Ababa University, Addis Ababa, Ethiopia;; ^15^ Department of Epidemiology and Biostatistics, Muhimbili University of Health and Allied Sciences, Dar es Salaam, Tanzania;; ^16^ Division of Public Health Institutes and Research, Africa Centres for Disease Control and Prevention, Addis Ababa, Ethiopia;; ^17^ University of Ibadan Research Foundation, University of Ibadan, Ibadan, Nigeria;; ^18^ Department of Nutrition, Harvard T.H. Chan School of Public Health, Harvard University, Boston, Massachusetts;; ^19^ Department of Epidemiology, Harvard T.H. Chan School of Public Health, Harvard University, Boston, Massachusetts

## Abstract

The COVID-19 pandemic has had serious negative health and economic impacts in sub-Saharan Africa. Continuous monitoring of these impacts is crucial to formulate interventions to minimize the consequences of COVID-19. This study surveyed 2,829 adults in urban and rural sites among five sub-Saharan African countries: Burkina Faso, Ethiopia, Nigeria, Tanzania, and Ghana. Participants completed a mobile phone survey that assessed self-reported sociodemographics, COVID-19 preventive practices, psychological distress, and barriers to healthcare access. A modified Poisson regression model was used to estimate adjusted prevalence ratios (aPRs) and 95% CIs to investigate potential factors related to psychological distress and barriers to reduced healthcare access. At least 15.6% of adults reported experiencing any psychological distress in the previous 2 weeks, and 10.5% reported that at least one essential healthcare service was difficult to access 2 years into the pandemic. The majority of participants reported using several COVID-19 preventive methods, with varying proportions across the sites. Participants in the urban site of Ouagadougou, Burkina Faso (aPR: 2.29; 95% CI: 1.74–3.03) and in the rural site of Kintampo, Ghana (aPR: 1.68; 95% CI: 1.21–2.34) had a higher likelihood of experiencing any psychological distress compared with those in the rural area of Nouna, Burkina Faso. Loss of employment due to COVID-19 (aPR: 1.77; 95% CI: 1.47–2.11) was also associated with an increased prevalence of psychological distress. The number of children under 5 years in the household (aPR: 1.23; 95% CI: 1.14–1.33) and participant self-reported psychological distress (aPR: 1.83; 95% CI: 1.48–2.27) were associated with an increased prevalence of reporting barriers to accessing health services, whereas wage employment (aPR: 0.67; 95% CI: 0.49–0.90) was associated with decreased prevalence of reporting barriers to accessing health services. Overall, we found a high prevalence of psychological distress and interruptions in access to healthcare services 2 years into the pandemic across five sub-Saharan African countries. Increased effort and attention should be given to addressing the negative impacts of COVID-19 on psychological distress. An equitable and collaborative approach to new and existing preventive measures for COVID-19 is crucial to limit the consequences of COVID-19 on the health of adults in sub-Saharan Africa.

## INTRODUCTION

COVID-19 is a viral acute respiratory infection caused by SARS COV2. The COVID-19 pandemic has culminated in a socioeconomic and health crisis on a global scale.^1^^–^^4^ COVID-19 has had a lasting impact on economies and livelihoods across the world, with a persisting burden of morbidity and mortality.^5^^,^^6^ As of July 17, 2022, more than 12 million COVID-19 cases and 256,414 deaths were reported on the African continent.^7^^,^^8^ Several vaccines have been approved for use in the general community to prevent severe morbidity and mortality from COVID-19.^9^^,^^10^ However, only 19.4% of the population in sub-Saharan Africa is fully vaccinated, in contrast to 66.8% in developed countries in July 2022.^11^ COVID-19 is still projected to remain a challenge for sub-Saharan African countries (SSA). In addition, fragile health systems, poverty, and other endemic illnesses further exacerbate the health and socioeconomic consequences of COVID-19 in SSA.^12^^,^^13^

Community perceptions of COVID-19 and preventive practices are important factors that can be addressed to decrease community transmission, as outlined by the WHO and several African health ministries.^14^^,^^15^ A previous study in SSA showed that adults reported low adherence to COVID-19 preventive measures (such as avoiding social gatherings and disinfecting contaminated surfaces), despite a good level of knowledge of effective preventive measures.^16^ Continuous monitoring of trends in COVID-19 prevention is necessary to increase adherence to preventive measures in future pandemics and to prioritize resource allocation in prevention campaigns.^17^^,^^18^

Although the negative impacts of COVID-19, such as reduced access and disruption of healthcare services, financial insecurities, and psychological distress, may have improved since the pandemic’s start, challenges persist in most low- and middle-income countries.^19^ Mental health challenges including psychological distress have been reported during the pandemic, likely due to increased isolation, continued inflation, job instability, substance use, poor health service provision, and economic instability.^20^^–^^23^

Moreover, COVID-19 has also affected access to routine healthcare due to government mitigation measures such as travel bans and lockdowns, patients’ fear of infection at health centers, and supply chain challenges.^24^^,^^25^ Several previous studies early in the pandemic (in 2020) reported that substantial proportions of adults in SSA faced difficulty in accessing routine maternal and childcare services due to fear of contracting COVID-19, lockdowns, and service disruptions.^26^^–^^29^ However, it is unclear whether these disruptions have persisted as the pandemic has progressed and which factors are contributing to this disruption in access to health services in SSA.

This study assessed COVID-19 perceptions and preventive practices, psychological distress, and disruption of health services during the COVID-19 pandemic across five sub-Saharan African countries: Nigeria, Ethiopia, Burkina Faso, Tanzania, and Ghana. Building off a baseline cross-sectional study conducted in 2020 among adult community members in urban and rural areas in Nigeria, Ethiopia, and Burkina Faso, this second survey round also included new sites in Tanzania and Ghana. This study will help shed light on trends in COVID-19 impacts and compliance with preventive measures, which is necessary for formulating and prioritizing preventive strategies to mitigate the consequences of the pandemic in SSA.

## MATERIALS AND METHODS

### Study population.

This survey was conducted by the Africa Research, Implementation Science, and Education (ARISE) Network, a research and training platform including 21 member institutions from nine sub-Saharan African countries. This study was a second-round mobile phone survey building on a 2020 baseline survey that was conducted in three ARISE countries (Ethiopia, Nigeria, and Burkina Faso). The study rationale, sampling strategies, and survey methodology for the baseline survey have been described in detail previously.^30^

Briefly, the first survey round included one urban and one rural site in three SSA countries: Nouna (rural) and Ouagadougou (urban) in Burkina Faso, Kersa (rural) and Addis Ababa (urban) in Ethiopia, and Ibadan (rural) and Lagos (urban) in Nigeria. The second survey round included a rural (Dodoma) and urban site (Dar es Salaam) in Tanzania and a site in Ghana (Kintampo), which is a largely rural area. The second survey round included a total of nine sites across five countries (Supplemental Figure 1). ARISE Network sites were selected for this survey based on the available data collection infrastructure, research capacity, and willingness of site leaders to take on the survey. The Round 2 survey was conducted between July and December 2021. The current study focuses on data collected from adult community members in the second survey round. The survey also collected data from adolescents and healthcare workers, but the results from those populations are presented elsewhere.

### Study design and sampling.

**Figure 1.**
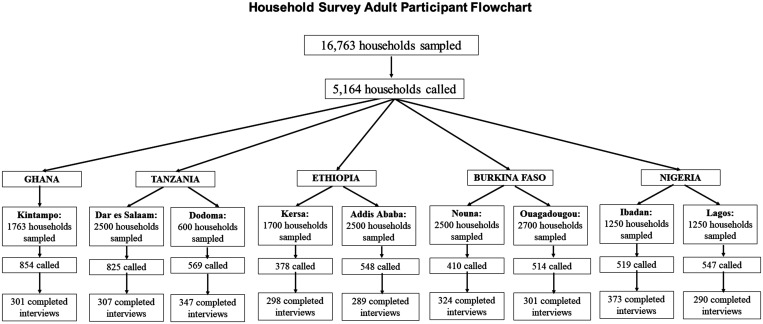
Africa Research, Implementation Science, and Education COVID-19 Survey Round 2 participant flowchart for the adult household survey across five countries, 2021.

Where possible, each site used health and demographic surveillance systems or existing national surveys to construct sampling frames. Health and Demographic Surveillance Systems were used in Burkina Faso, rural Ethiopia (Kersa), Tanzania, and Ghana. In Nigeria, the National Living Standard Survey 2017–2018 and lists from telephone service providers were used. In Addis Ababa, no existing surveys were available before the round 1 survey; therefore, we conducted a household survey in round 1 and used this as the sampling frame in round 2. In each household, one adult aged 20 or older was interviewed. Because this was a phone survey, participants were limited to those who had access to a working phone (mobile or landline). Households were sampled from each sampling frame and called until we reached the target sample size of 300 for each site (600 per country except for Ghana, which only included 300 adults from a rural area). Figure 1 shows the number of participants sampled, called, and interviewed at each site.

All adults who participated in the round 1 survey in Burkina Faso, Ethiopia, and Nigeria were recontacted to join this survey. We replaced those adults who could not be reached or who declined to participate in the study with new participants from the sampling frame to achieve the target sample size of 300 adults per site. In Tanzania and Ghana, all recruited participants were new because these countries did not participate in the round 1 survey.

### Data collection.

Trained enumerators conducted all interviews in the local languages of each site using computer-assisted telephone interviewing (CATI). Participant data were collected electronically using a mobile tablet-based data collection system (Open Data Kit) and uploaded to a secure server after each interview. All research staff members were trained on study procedures, including screening, consent, enrollment, and data collection, emphasizing confidentiality and safeguarding the participant’s rights and well-being. A standardized questionnaire assessing sociodemographic information; knowledge, practices, and perceptions of COVID-19; psychological distress; reported disruption of health services due to COVID-19; and knowledge, perceptions, beliefs, and hesitancy related to COVID-19 vaccines was used across all sites, which was developed by subject matter experts across sites. Experts familiar with the context of each site translated the consent script and the questionnaires into the local languages of each country. The questionnaire was pretested at each site, and minor adaptations were made to account for the local contexts in each area. The complete questionnaire is available at (https://africa.harvard.edu/covid-19-resources). The data collectors obtained verbal informed consent from each participant before beginning the interview. Data were reviewed for completeness and quality, cleaned, and pooled for data analysis by a centralized data management team.

### Ethical approval.

Informed consent was obtained from all participants in this survey and the study was approved by all necessary ethical review boards in each country, including the Harvard T.H. Chan School of Public Health Institutional Review Board; Nouna Health Research Center Ethical Committee, and National Ethics Committee in Burkina Faso, the Institutional Ethical Review Board of Addis Continental Institute of Public Health in Ethiopia, Kintampo Health Research Center Institutional Ethics Committee in Ghana, the University of Ibadan Research Ethics Committee in Nigeria, and the Muhimbili University of Health and Allied Sciences, University of Dodoma and the National Institute for Medical Research in Tanzania.

### Data analysis.

Survey responses to questions related to sociodemographics, COVID-19 practices and perceptions, psychological distress, and disruption of healthcare access due to COVID-19 were presented descriptively. Means and SDs were presented for continuous variables, medians, and interquartile ranges for skewed variables, and counts and percentages for categorical variables. Demographic characteristics explored included age, gender, occupation, religion, household size, number of children under 5 years, and participant’s role in the household (mother, father, or other). Participants were also asked if they considered themselves to be the head of the household which in this study context referred to the individual who served as the provider of the family (often a senior male in the home).^31^ Household wealth was defined using a wealth index, constructed using principal component analysis of 10 items describing the household’s asset ownership, housing quality, crowding, and water and sanitation facilities. The wealth index was divided into population tertiles (poor, middle, and rich).^32^^,^^33^

Perceptions and compliance with preventive measures were assessed by asking participants if they complied with the following evidence-based prevention measures: use of masks, handwashing, changing travel plans, and social distancing. In addition, we also assessed some non–evidence-based perceptions and preventive measures such as utilizing saunas, taking vitamins, and drinking tea or consuming ginger as preventive measures for COVID-19.

We measured psychological distress using the validated four-item Patient Health Questionnaire for Depression and Anxiety Scale (PHQ-4),^34^^,^^35^ which included four questions related to psychological distress over the past two weeks. This instrument asks participants how often they have been bothered by the following problems over the past 2 weeks: 1) feeling nervous, anxious, or on edge; 2) not being able to control or stop worrying; 3) having little interest or pleasure in doing things; 4) feeling down, depressed, or hopeless. Each question is answered on a scale where 0 = not at all, 1 = several days, 2 = more than half the days, and 3 = nearly every day. We computed a total score for psychological distress by adding the scores of the four items, ranging from 0 to 12. We further categorized psychological distress as none (total score: 0–2), mild (total score: 3–5), moderate (total score: 6–8), severe (total score: 9–12), and any psychological distress (including mild, moderate, and severe).^35^ We also created an anxiety subscale (range: 0–6) using the scores of the first two questions and a depression subscale (range: 0–6) using the scores of the last two questions. A subscale score of 3 or greater was considered a high anxiety or depression score.^34^ The PHQ-4 has previously been proven valid and reliable for screening for anxiety and depression among adolescents and adults in sub-Saharan Africa.^35^

We also examined access to seven essential health services as reported by the adult community participants. Participants were asked if COVID-19 impacted their access to the following healthcare services: 1) childhood immunization, 2) vitamin A supplementation for children, 3) management of child malnutrition, 4) antenatal care for pregnant women, 5) iron and folic acid supplementation, 6) sexual and reproductive health services, and 7) HIV treatment services. The first three services were grouped as child health services and the second three as maternal and reproductive health services. For each question, participants could respond with yes (scored as 1 point), no (scored as 0 points), not applicable, don’t know, or refuse to answer. Responses were coded as missing and unscored if the services were not applicable or participants refused to answer the question. Answers to each question were summed across all sites to create a total aggregate score. The maximum score for child health services was 3 (1 point each for immunization, malnutrition treatment, and vitamin A). The maximum score for maternal and reproductive health was 3 (1 point each for antenatal care, iron and folic acid supplementation, and sexual and reproductive health services). The remainder, HIV services, had a maximum point of one. A total aggregated score was computed by summing responses to all seven health services questions, ranging from 0 to 7. Each site’s mean total aggregated score was used as a cutoff point to define reduced access to essential health services (below the mean aggregated score indicated reported difficulty in accessing healthcare).^36^^,^^37^ The respective cutoff for Nouna, Ouagadougou, Addis Ababa, Kersa, Ibadan, Lagos, Dar es Salaam, Dodoma, and Kintampo was 0.041, 0.707, 0.211, 0.184, 1.793, 0.603, 0.035, 0.006, and 0.269, respectively.

In associational analyses, our primary outcomes of interest were reduced access to essential health services and any self-reported psychological distress, which was selected based on its higher prevalence (19.9%) compared with other psychological distress outcomes (≤ 10%). Potential factors explored for each outcome were site, age, gender, occupation, role in the household, educational status, wealth index, COVID-19 testing availability, ever tested positive for COVID-19, the number of children under 5 years in the household, household size, and effect of COVID-19 on employment. Any psychological distress was also explored as a factor relating to reduced healthcare access and vice versa. Because of the low reported difficulty of healthcare access in Dar es Salaam, Dodoma, Nouna, and Kersa, these sites were excluded in the crude and adjusted analyses for this outcome (reduced access). Modified Poisson regression with robust standard errors^38^ was used to estimate crude prevalence ratios and adjusted prevalence ratios (aPRs) with 95% CIs and *P* values to establish statistical significance (α < 0.05). In the crude analysis, variables with a *P* value < 0.25 were retained for the final model. However, age and gender had *P* values > 0.25 but were kept in the model because the authors hypothesized these factors were essential to include. Model fitness and collinearity effects were tested using model misspecification and correlation matrices. Missing data were handled using a complete case analysis. Data were cleaned and managed using SAS 9.4 (SAS Institute Inc., Cary, NC) and analyzed using Stata version 16 (Stata Corp LLC, College Station, TX).

## RESULTS

### Sociodemographic characteristics.

A total of 2,829 adults from rural and urban sites in Burkina Faso (*N* = 624), Ethiopia (*N* = 587), Nigeria (*N* = 663), Tanzania (*N* = 654), and a rural location in Ghana (*N* = 301) participated in the study. The majority of the participants in Burkina-Faso (62.5%) and Ethiopia (59.3%) previously participated in the round 1 survey, whereas less than half did in Nigeria (46.4%). The median age of the participants was 41 years, and the rural site of Ethiopia (Kersa) had the youngest mean age across all sites (20 years). Across all sites, less than half of the study participants were female (43.5%). However, the majority were female in the urban site in Ethiopia (Addis Ababa (69.0%)) and the rural site in Tanzania (Dodoma (67.2%)). The majority of the respondents identified themselves as the head of the household (66.0%). Across all sites, 23% of participants had no education, and 34% had completed some primary schooling. The median number of household members and children under 5 years old present in the household were six and one, respectively. Even though most of the adults across the sites reported the COVID-19 pandemic did not affect their job status, half of the participants reported that their income was affected (Table 1).

**Table 1:** Sociodemographic and other characteristics of adult community members in a phone-based survey in five sub-Saharan African countries, 2021*

	Burkina Faso		Ethiopia		Nigeria		Tanzania		Ghana	Total
	Urban	Rural	Urban	Rural	Urban	Rural	Urban	Rural	Rural	
	Ouagadougou	Nouna	Addis	Kersa	Lagos	Ibadan	Dar es Salaam	Dodoma	Kintampo	
Adults, *n*	300	324	289	298	290	373	307	347	301	2,829
Adults who also participated in the round 1 survey, *n*	175 (58.33)	215 (66.36)	167 (57.78)	181 (60.74)	161 (55.52)	147 (39.41)	NA	NA	NA	1,046 (55.82)
Age, *n* (%), years										
20–29	8 (2.66)	15 (4.63)	72 (24.91)	66 (22.15)	55 (19.97)	88 (23.59)	16 (5.21)	32 (9.22)	76 (25.25)	428 (15.12)
30–39	52 (17.28)	63 (20.37))	112 (38.75)	166 (38.93)	99 (34.14)	91 (24.40)	53 (17.26)	96 (27.67)	79 (26.25)	764 (27.00)
40–49	124 (41.20)	120 (37.04)	51 (17.65)	76 (22.50)	85 (29.31)	105 (28.15)	106 (34.53)	130 (37.46)	83 (27.57)	880 (31.10)
≥ 50	116 (37.67)	123 (37.96)	54 (18.69)	40 (13.42)	51 (17.59)	89 (23.86)	132 (43.00)	89 (25.65)	63 (20.93)	757 (26.76)
Summary statistics†	47 (41.00; 54.00)	45 (39.50; 55.00)	35 (30.00; 45.00)	35 (30.00; 44.00)	38 (31.00; 45.00)	40 (30.00; 49.00)	47 (40.00; 57.00)	43 (35.00; 50.00)	39 (29.00; 48.00)	41 (33.0; 50.00)
Gender, *n* (%)										
Female	103 (34.33)	50 (15.43)	202 (69.90)	44 (14.77)	131 (45.17)	164 (43.97)	146 (47.56)	232 (66.86)	160 (53.16)	1,232 (43.55)
Male	197 (65.67)	274 (84.57)	87 (30.10)	254 (85.23)	159 (54.83)	209 (56.03)	161 (52.44)	115 (33.14)	141 (46.84)	1,579 (56.45)
Head of the household, *n* (%)	237 (79.0)	244 (75.3)	211 (73.0)	265 (88.9)	155 (53.4)	187 (50.1)	224 (73.0)	184 (53.0)	161 (53.5)	1,868 (66.0)
Role in the household										
Father/husband	189 (63.00)	259 (79.94)	79 (27.34)	247 (82.88)	132 (45.52)	132 (35.39)	153 (49.84)	114 (32.85)	111 (36.88)	1,452 (51.33)
Mother/wife	106 (35.33)	47 (14.51)	173 (59.86)	46 (15.44)	102 (35.17)	168 (45.04)	146 (47.55)	228 (65.71)	127 (42.19)	1,107 (39.13)
Other‡	5 (1.67)	18 (5.55)	37 (12.80)	5 (1.68)	56 (19.31)	73 (19.57)	8 (2.61)	5 (1.44)	63 (20.93)	270 (9.54)
Highest level of education, *n* (%)										
None	172 (57.33)	179 (55.25)	32 (11.07)	82 (27.52)	5 (1.72)	10 (2.68)	10 (3.26)	79 (22.77)	91 (30.23)	660 (23.33)
Primary	68 (22.67)	105 (32.41)	80 (27.68)	156 (52.35)	11 (3.79)	38 (10.2)	175 (57.00)	252 (72.62)	76 (25.25)	961 (33.97)
Secondary	48 (16.00)	27 (8.33)	106 (36.68)	41 (13.76)	48 (16.55)	77 (20.6)	92 (29.97)	16 (4.61)	100 (33.22)	555 (19.62)
Tertiary and higher	12 (4.00)	13 (4.01)	71 (24.57)	19 (6.37)	226 (77.93)	248 (66.5)	30 (9.77)	0 (0.00)	34 (11.30)	653 (23.08)
Occupation, *N* (%)§										
Unemployed	25 (8.33)	0 (0.00)	59 (20.42)	0 (0.00)	4 (1.38)	13 (3.49)	30 (9.77)	2 (0.58)	31 (10.30)	164 (5.80)
Student	2 (0.67)	2 (0.62)	2 (0.69)	9 (3.02)	26 (8.97)	59 (15.82)	1 (0.33)	0 (0.00)	11 (3.65)	112 (3.96)
Farmer	15 (5.00)	235 (72.53)	0 (0.00)	268 (89.93)	4 (1.38)	7 (1.88)	22 (7.17)	333 (95.97)	119 (39.53)	1,003 (35.45)
Wage employment	35 (11.67)	13 (4.01)	60 (20.76)	15 (5.03)	103 (35.52)	100 (26.81)	49 (15.96)	4 (1.15)	46 (15.28)	425 (15.02)
Self-employed	130 (43.33)	31 (9.57)	107 (37.02)	10 (3.36)	117 (40.34)	129 (34.58	172 (56.03)	6 (1.73)	118 (39.20)	820 (28.99)
Other‖	47 (15.67)	35 (10.80)	63 (21.80)	26 (8.72)	1 (0.34)	6 (1.61)	36 (11.73)	20 (5.76)	8 (2.66)	242 (8.55)
Household size†	7 (6.00; 9.00)	9 (7.00; 13.00)	4 (3.00; 6.00)	7 (5.00; 8.00)	4 (3.00; 5.00)	5 (3.00; 6.00)	5 (4.00; 7.00)	6 (5.00; 8.00)	6 (5.00; 9.00)	6 (4.00; 8.00)
No. of children aged < 5 years†	1 (0.00; 1.00)	1 (0.00; 2.00)	0 (0.00; 1.00)	1 (0.00; 2.00)	0 (0.00; 1.00)	0 (0.00; 1.00)	0 (0.00; 1.00)	0 (0.00; 1.00)	1 (0.00; 2.00)	1 (0.00; 1.00)
Wealth index¶										
Poor	100 (33.33)	117 (36.11)	97 (33.56)	83 (27.85)	39 (13.64)	120 (32.26)	90 (29.61)	130 (37.46)	101 (33.55)	877 (31.09)
Middle	101 (33.67)	101 (31.17)	177 (61.25)	113 (37.92)	144 (50.35)	125 (33.60)	117 (38.49)	155 (44.67)	111 (36.88)	1,144 (40.55)
Rich	99 (33.00)	106 (32.72)	15 (5.19)	102 (34.23)	103 (36.01)	127 (34.14)	97 (31.91)	62 (17.87)	89 (29.57)	800 (28.36)
How COVID-19 affected respondent’s job#										
No change	199 (66.33)	288 (88.89)	228 (78.89)	280 (93.96)	242 (83.45)	291 (78.02)	160 (62.50)	330 (95.10)	268 (89.04)	2,286 (82.29)
Lost employment	69 (23.00)	34 (10.49)	46 (15.92)	2 (0.67)	15 (5.17)	43 (11.53)	10 (3.91)	6 (1.73)	15 (4.98)	240 (8.64)
Changed employment	32 (10.67)	2 (0.62)	15 (5.19)	16 (5.37)	33 (11.38)	39 (10.45)	86 (33.59)	11 (3.17)	18 (5.98)	252 (9.07)
How COVID-19 affected your income**										
No change	88 (29.33)	199 (61.42)	138 (47.75)	243 (81.54)	107 (36.90)	156 (41.82)	99 (40.91)	125 (77.64)	104 (34.55)	1,259 (48.84)
Lost/reduced income	211 (70.33)	125 (38.58)	131 (45.33)	54 (18.12)	172 (59.31)	203 (54.42)	139 (57.44)	36 (22.36)	194 (64.45)	1,265 (49.07)
Increased salary/ income	1 (0.33)	0 (0.00)	20 (6.92)	1 (0.34)	11 (3.79)	14 (3.76)	4 (1.65)	0 (0.00)	3 (1.00)	54 (2.09)

*n* (%) = number of observations (percentage); *n* = number of observations; NA = not applicable.

* Number of observations (percentage).

† Median (25th; 75th percentile).

‡ Uncle, sister, or aunt.

§ Counts and percentages do not add up to 100% because multiple responses were allowed.

‖ Casual, teacher, preacher, stay-at-home, welder, police officer, journalist, lawyer, builder.

¶ One, four, and three observations were missing in Ibadan, Lagos, and Dar es Salaam respectively.

# 51 observations were missing in Dar es Salaam.

** In Dodoma and Dar es Salaam, 186 and 65 observations were missing, respectively.

### COVID-19 preventive measures and testing availability.

A small proportion of participants in Kintampo, Ghana (1.3%) and across the rest of the sites (4.1%) did not use any preventive methods to reduce their risk of acquiring COVID-19 infection. Across all sites, the majority of participants reported regular handwashing (89.6%) and wearing masks (85.0%). The rural site in Tanzania (Dodoma) had the smallest (24.2%), and the urban site in Burkina Faso (Ouagadougou) had the highest (90%) proportion of adults reporting social distancing as a COVID-19 preventive measure. However, the urban sites in Tanzania (Dar es Salaam), Ethiopia (Addis Ababa), and Nigeria (Lagos) had the highest proportion of adults reporting using misinformed prevention measures such as steaming or saunas (23.1%), drinking lemon or consuming ginger (45.0%), and taking nutritional supplements (57.6%) to prevent COVID-19. COVID-19 testing was reportedly available for 38% of participants overall; the highest availability was in the urban site of Addis Ababa, Ethiopia (85%), and the lowest was in the rural area of Kersa, Ethiopia (4%). Among participants who reported COVID-19 testing was available, 81% reported that it was free of charge. Across all sites, 16.3% and 16.5% of participants had ever tested for COVID-19 and knew anyone who had been sick from COVID-19, respectively (Table 2).

**Table 2:** COVID-19 preventive practices and psychological distress outcomes among adult community members in a phone-based survey in five sub-Saharan African countries, 2021*

	Burkina Faso		Ethiopia		Nigeria		Tanzania		Ghana	Total
	Urban	Rural	Urban	Rural	Urban	Rural	Urban	Rural	Rural	
	Ouagadougou	Nouna	Addis	Kersa	Lagos	Ibadan	Dar es Salaam	Dodoma	Kintampo	
Adults, *n*	300	324	289	298	290	373	307	347	301	2,829
Methods taken to reduce risk of transmission†										
Nothing	1 (0.33)	23 (7.10)	1 (0.35)	65 (21.81)	0 (0.00)	0 (0.00)	5 (1.63)	17 (4.90)	4 (1.33)	116 (4.10)
Handwashing with soap and/or use hand sanitizer	297 (99.00)	294 (90.74)	268 (92.73)	177 (59.40)	279 (96.21)	361 (96.78)	273 (88.93)	298 (85.88)	289 (96.01)	2,536 (89.64)
Social distancing	270 (90.00)	249 (76.85)	125 (43.25)	162 (54.63)	241 (83.10)	281 (75.34)	86 (28.01)	84 (24.21)	233 (77.41)	1,731 (61.19)
Wear face mask	296 (98.67)	268 (82.72)	281 (97.23)	217 (72.82)	274 (94.48)	350 (93.83)	259 (84.36)	170 (48.99)	289 (96.01)	2,404 (84.98)
Cancel/change travel plans	41 (13.67)	91 (28.09)	13 (4.50)	3 (1.01)	20 (6.90)	19 (5.09)	7 (2.28)	22 (6.34)	78 (25.91)	294 (10.39)
Drink/eat lemon, ginger/ garlic	28 (9.33)	39 (12.04)	130 (44.98)	48 (16.11)	98 (33.79)	94 (25.20)	85 (27.69)	77 (22.19)	93 (30.90)	692 (24.46)
Take nutritional supplements	1 (0.33)	1 (0.31)	81 (28.03)	43 (14.43)	167 (57.59)	164 (43.97)	2 (0.65)	2 (0.58)	46 (15.28)	507 (17.92)
Steaming or sauna	17 (5.67)	3 (0.93)	7 (2.42)	0 (0.00)	5 (1.72)	6 (1.61)	71 (23.13)	14 (4.03)	21 (6.98)	144 (5.09)
COVID-19 testing available	74 (24.67)	178 (54.94)	246 (85.12)	12 (4.03)	162 (55.86)	194 (52.01)	91 (29.64)	46 (13.26)	70 (23.26)	1,073 (37.93)
If yes, free or paid testing‡										
Free	50 (67.57)	166 (93.26)	244 (99.19)	12 (100.00)	95 (58.64)	137 (70.62)	80 (87.91)	40 (86.96)	45 (64.29)	869 (80.99)
Paid	2 (2.70)	0 (0.00)	2 (0.81)	0 (0.00)	29 (17.90)	33 (17.01)	2 (2.20)	1 (2.17)	4 (5.71)	73 (6.80)
Don’t know	22 (29.73)	12 (6.74)	0 (0.00)	0 (0.00)	38 (23.46)	24 (12.37)	9 (9.89)	5 (10.87)	21 (30.00)	131 (12.21)
Ever been tested for COVID-19§	18 (5.98)	46 (14.20)	165 (57.09)	1 (0.34)	57 (19.66)	85 (22.79)	46 (14.98)	5 (1.44)	39 (12.96)	462 (16.33)
Ever tested positive for COVID-19‖	0 (0.00)	3 (6.52)	21 (12.73)	1 (100.00)	14 (24.56)	5 (5.88)	3 (6.52)	1 (20.00)	1 (2.56)	49 (10.61)
Knew anyone who was sick from COVID-19	11(3.67)	10 (3.09)	164 (56.75)	8 (2.68)	92 (31.72)	86 (23.06)	70 (22.80)	19 (5.48)	7 (2.33)	467 (16.51)
Psychological distress outcomes										
Mild psychological distress¶	112 (37.33)	34 (10.49)	39 (13.49)	36 (12.08)	33 (11.50)	55 (14.75)	32 (10.42)	34 (9.80)	66 (21.93)	441 (15.62)
Moderate psychological distress¶	15 (5.00)	20 (6.17)	14 (4.84)	2 (0.67)	13 (4.53)	8 (2.14)	8 (2.61)	1 (0.29)	8 (2.68)	89 (3.15)
Severe psychological distress¶	2 (0.67)	4 (1.23)	4 (1.38)	0 (0.00)	7 (2.44)	8 (2.14)	2 (0.65)	0 (0.00)	3 (1.00)	30 (1.06)
Any psychological distress¶	129 (43.00)	58 (17.90)	57 (19.72)	38 (12.75)	53 (18.47)	71 (19.03)	42 (13.68)	35 (10.09)	77 (25.75)	560 (19.83)
High anxiety score#	24 (8.00)	27 (8.33)	28 (9.69)	7 (2.35)	28 (9.76)	23 (6.17)	12 (3.91)	3 (0.86)	28 (9.36)	180 (6.37)
High depression score#	25 (8.33)	23 (7.17)	23 (7.96)	3 (1.01)	11 (3.83)	25 (6.70)	9 (2.93)	2 (0.58)	13 (4.35)	134 (4.75)

*n* (%) = Number of observations (percentage); *n* = number of observations.

* Number of observations (percentage).

† Counts and percentages do not add up to the total due to selecting multiple responses.

‡ 1,075 total observations.

§ 2,830 total observations.

‖ From 404 total observations.

¶ Psychological distress was measured using the four-item Patient Health Questionnaire for Depression and Anxiety Scale. Each item had a numeric score of 0, 1, 2, and 3, and the total score was computed by adding up the scores of the four items, with a range of 0–12. The total score of psychological distress was categorized into none (total score: 0–2), mild (total score: 3–5), moderate (total score: 6–8), and severe (total score: 9–12), a total of 5 observations, 3 in Lagos and 2 in Kintampo are missing.

# An anxiety subscale (range: 0–6) and a depression subscale (range: 0–6) were created using the scores of the first and last two questions, respectively; a subscale score of 3 or greater was considered a high level of anxiety and depression, respectively; 5 observations, 3 in Lagos and 2 in Kintampo are missing.

### Psychological distress and COVID-19.

Across all sites, nearly 20% and 16% of participants had any psychological or mild psychological distress, respectively. The urban location of Burkina Faso (Ouagadougou) and the rural area of Ghana (Kintampo) had the highest proportions of mild psychological distress (37.3% and 21.9%, respectively), compared with the other sites. The urban sites in Ethiopia (Addis Ababa) and Tanzania (Dar es Salaam) had higher reported prevalence for all types of psychological distress than their rural counterparts. However, the rural sites in Burkina Faso (Ouagadougou) and Nigeria (Ibadan) had higher reported moderate, severe, and mild psychological outcomes than their urban counterparts. Across sites, a small proportion of adults reported moderate (3.2%) and severe psychological distress (1.1%). The rural site of Dodoma, Tanzania, had the lowest proportion of all types of psychological distress outcomes across all sites. Approximately 6.4% and 4.8% of all participants across the sites had self-reported anxiety and depression (Table 2).

### Self-reported impact of COVID-19 on accessing healthcare services.

**Figure 2.**
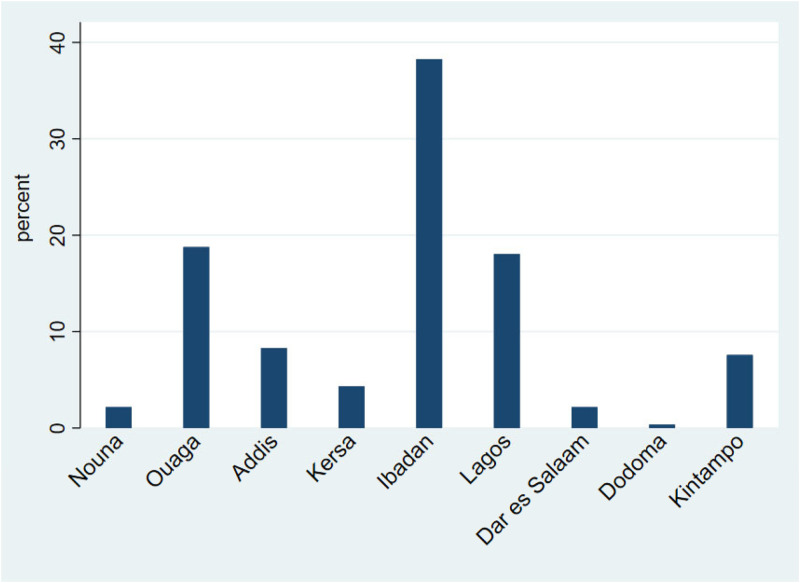
The proportion of adults reporting interruptions in essential health services due to the COVID-19 pandemic by site among nine urban and rural sites in five sub-Saharan African countries, December 2021.

Across all sites, the percentage of adults reporting that the COVID-19 pandemic had affected their or their family’s access to health services was 7.8% for children’s immunization, 7.4% for vitamin A supplementation, and 6% for malnutrition management services. Tanzanian adults and those in Nouna, Burkina Faso, and Kersa, Ethiopia, had the lowest reported proportion of interrupted access to all three types of child health services during the pandemic (< 2.8%). Across all sites, 7.4% of participants reported difficulty accessing antenatal care due to the COVID-19 pandemic, 6.1% reported difficulty accessing folic acid supplementation, and 6.8% reported problems accessing family planning. The mean aggregated score for reported interrupted access to the child, maternal, and total health services was 0.21, 0.19, and 0.44, respectively. Across sites, participants from the rural site in Nigeria (Ibadan), followed by the urban sites in Burkina Faso (Ouagadougou) and Nigeria (Lagos), had the highest proportion of reported interruption of healthcare access across the seven types of health services (Figure 2). Across healthcare services, HIV treatment had the lowest proportion of reported disruption (5.4%), and children’s immunization services had the highest proportion of reported interruption (7.8%) (Table 3).

**Table 3:** Interruption in access to health services due to the COVID-19 pandemic from the perspective of adult community members in a phone-based survey in five sub-Saharan African countries, 2021

	Burkina Faso		Ethiopia		Nigeria		Tanzania		Ghana	Total
	Urban	Rural	Urban	Rural	Urban	Rural	Urban	Rural	Rural	
	Ouagadougou	Nouna	Addis	Kersa	Lagos	Ibadan	Dar es Salaam	Dodoma	Kintampo	
No. of adults	300	324	289	298	290	373	307	347	301	2,829
Childhood immunization*	287	307	259	288	267	313	281	343	199	2,544
Yes	41 (14.29)	2 (0.65)	13 (5.02)	8 (2.78)	27 (10.11)	89 (28.43)	3 (1.07)	0 (0)	16 (8.04)	199 (7.82)
No	246 (85.71)	305 (99.35)	246 (94.98)	280 (97.22)	240 (89.89)	224 (71.57)	278 (98.93)	343 (100.00)	183 (91.96)	2,345 (92.18)
Missing/not applicable	13	17	30	10	23	60	26	4	102	285
Vitamin A supplementation for children*	286	295	251	287	264	307	281	345	199	2,515
Yes	37 (12.94)	3 (1.02)	14 (5.58)	8 (2.79)	24 (9.09)	86 (28.01)	2 (0.71)	0 (0.00)	13 (6.53)	187 (7.44)
No	249 (87.06)	292 (98.98)	237 (94.42)	279 (97.21)	240 (90.91)	221 (71.99)	279 (99.29)	345 (100.00)	186 (93.47)	2,328 (92.56)
Missing/not applicable	14	29	38	11	26	66	26	2	102	314
Management of child malnutrition*	287	279	232	285	260	304	285	346	187	2,465
Yes	14 (4.88)	3 (1.08)	8 (3.45)	7 (2.46)	21 (8.08)	79 (25.99)	1 (0.35)	1 (0.29)	14 (7.49)	148 (6.00)
No	273 (95.12)	276 (98.92)	224 (96.55)	278 (97.54)	239 (91.92)	225 (74.01)	284 (99.65)	345 (99.71)	173 (92.51)	2,317 (94.00)
Missing/not applicable	13	45	57	13	30	69	22	1	114	364
Child health access difficulty score†	0.32 (0.79)	0.03(0.27)	0.13 (0.53)	0.08 (0.48)	0.27 (0.78)	0.80 (1.27)	0.02 (0.22)	0.003 (0.05)	0.21 (0.74)	0.21 (0.71)
Antenatal care for pregnant women*	288	299	239	287	256	305	283	347	119	2,423
Yes	40 (13.89)	2 (0.67)	5 (2.09)	8 (2.79)	27 (10.55)	90 (29.51)	0 (0.00)	0 (0.00)	7 (5.88)	179 (7.39)
No	248 (86.11)	297 (99.33)	234 (97.91)	279 (97.21)	229 (89.45)	215 (70.49)	283 (100.00)	347 (100.00)	112 (94.12)	2,244 (92.62)
Missing/not applicable	12	25	50	11	34	68	24	0	182	406
Iron and folic acid for pregnant women*	284	287	231	282	258	306	284	347	123	2,402
Yes	19 (6.69)	1 (0.35)	6 (2.60)	9 (3.19)	19 (7.36)	86 (28.10)	0 (0.00)	0 (0.00)	6 (4.88)	146 (6.08)
No	265 (93.31)	286 (99.65)	225 (97.40)	273 (96.81)	239 (92.64)	220 (71.90)	284 (100.00)	347 (100.00)	117 (95.1)	2,256 (93.92)
Missing/not applicable	16	37	58	16	32	67	23	0	178	427
Family planning and reproductive health*	290	289	245	276	259	301	285	345	193	2,483
Yes	41 (14.14)	1 (0.35)	6 (2.45)	8 (2.90)	29 (11.20)	78 (25.91)	1 (0.35)	1 (0.29)	5 (2.59)	170 (6.85)
No	249 (85.86)	288 (99.65)	239 (97.55)	268 (97.10)	230 (88.80)	223 (74.09)	284 (99.65)	344 (99.71)	188 (97.41)	2,313 (93.15)
Missing/not applicable	10	35	44	22	31	72	22	2	108	346
Maternal and reproductive health access difficulty score‡	0.34 (0.84)	0.014 (0.14)	0.07 (0.34)	0.09 (0.46)	0.29 (0.76)	0.81 (1.27)	0.003 (0.06)	0.003 (0.05)	0.08 (0.45)	0.19 (0.68)
HIV treatment services*	286	209	214	256	261	298	283	347	121	2,275
Yes	16 (5.59)	1 (0.52)	4 (1.87)	6 (2.34)	17 (6.51)	73 (24.50)	3 (1.06)	0 (0.00)	4 (3.31)	124 (5.45)
No	270 (94.41)	208 (99.48)	210 (98.13)	250 (97.66)	244 (93.49)	225 (75.50)	280 (98.94)	347 (100.00)	117 (96.69)	2,151 (94.55)
Missing/not applicable	14	115	75	42	29	75	24	0	180	554
Total aggregated mean access difficulty score§	0.71 (1.72)	0.04 (0.36)	0.21 (0.83)	0.18 (1.03)	0.60 (1.63)	1.79 (2.87)	0.03 (0.26)	0.006 (0.11)	0.27 (1.04)	0.44 (1.49)
Reduced access to care (percentage)‖	52/293 (17.75)	6/319 (1.88)	23/265 (8.68)	12/294 (4.08)	50/272 (18.38)	106/324 (32.72)	6/289 (2.08)	1/347 (0.29)	21/242 (8.68)	277/2,645 (10.47)

* Number of observations with nonmissing data for each question.

† Aggregated mean score (SD) for childhood immunization, vitamin A supplementation, and management of child nutrition services; participants could respond with yes (scored as 1 point), no (scored as 0 points), not applicable, don’t know or refuse to answer. Responses were coded as missing and unscored if the services were irrelevant or participants declined to answer the question. The scores of the three questions were summed across all sites to create the child health difficulty access score.

‡ Aggregated mean score (SD), for antenatal care, iron and folic acid for pregnant women, and family planning and reproductive health services. Participants could respond with yes (scored as 1 point), no (scored as 0 points), not applicable, don’t know, or refuse to answer. Responses were coded as missing and unscored if the services were irrelevant or participants declined to answer the question. The scores of the three questions were summed across all sites to create maternal health access difficulty scores.

§ Total aggregated mean or mean scores of reported access difficulty of all health services (SD). Calculated by summing all the scores of seven health services.

‖ Number of participants with health service interruption score above the average score (across all sites) divided by adults with nonmissing data and multiplied by 100% to get the percentage.

### Factors associated with any psychological distress.

Household size was associated with an increased prevalence of reporting any psychological distress (aPR for a one-unit increase in household size 1.02; 95% CI: 1.00–1.04). Adults in the urban site of Burkina Faso (Ouagadougou) and rural site in Ghana (Kintampo) were 2.29 (95% CI: 1.74–3.03) and 1.68 (95% CI: 1.21–2.34) times as likely to report experiencing any psychological distress, compared with rural site in Burkina Faso (Nouna). Participants who reported wage employment had a 25% lower prevalence (aPR: 0.75; 95% CI: 0.59–0.95) of reporting any psychological distress compared with their counterparts (students, farmers, self-used, and not used). Compared with adults with steady jobs during the pandemic, participants who lost their job had a higher prevalence of any psychological distress (aPR: 1.77; 95% CI: 1.47–2.11). In addition, adults who changed jobs due to COVID-19 had a higher prevalence (aPR: 1.82; 95% CI: 1.48–2.24) of experiencing any-psychological distress compared with adults with stable employment (Table 4).

**Table 4:** Factors associated with any psychological distress during the COVID-19 pandemic among 2,769 adults in five sub-Saharan African countries, 2021*

	Any psychological distress, *n* (%)†	CPR	95% CI	*P* value	aPR	95% CI	*P* value
Site							
Nouna, Burkina Faso	58 (10.3)	Ref	1	–	Ref	1	–
Ouagadougou, Burkina Faso	129 (23.0)	2.40	1.85–3.15	< 0.001	2.29	1.74–3.03	< 0.001¶
Addis Ababa, Ethiopia	57 (10.2)	1.10	0.79–1.53	0.564	1.25	0.86–1.83	0.238
Kersa, Ethiopia	38 (6.8)	0.71	0.49–1.04	0.078	0.87	0.58–1.31	0.502
Ibadan, Nigeria	71 (12.8)	1.06	0.78–1.45	0.701	1.37	0.93–2.03	0.110
Lagos, Nigeria	53 (9.5)	1.03	0.74–1.56	0.856	1.43	0.95–2.18	0.089
Dar es Salaam, Tanzania	42 (7.5)	0.76	0.53–1.10	0.149	0.84	0.55–1.30	0.448
Dodoma, Tanzania	35 (6.3)	0.56	0.38–0.83	0.004	0.69	0.45–1.06	0.093
Kintampo, Ghana	77 (13.8)	1.44	1.06–1.95	0.018	1.68	1.21–2.34	0.002¶
Education							
None	176 (31.4)	Ref	1	–	Ref	1	–
Primary	161 (28.7)	0.63	0.52–0.76	< 0.001	0.84	0.69–1.03	0.091
Secondary	111 (19.8)	0.75	0.60–0.92	0.006	0.82	0.65–1.04	0.099
Tertiary and higher	112 (20.0)	0.64	0.52–0.79	< 0.001	0.75	0.55–1.02	0.064
Household size	7.0 (3.6)	1.03	1.02–1.05	< 0.001	1.02	1.00–1.04	0.033¶
Wage employment							
No‡	494 (88.2)	Ref	1	–	–	1	–
Yes	66 (11.8)	0.76	0.60–0.95	0.019	0.75	0.59–0.95	0.019¶
COVID-19 effect on job§							
None	385 (69.5)	Ref	1	–	–	1	–
Lost employment	92 (16.6)	2.29	1.90–2.75	< 0.001	1.77	1.47–2.11	< 0.001¶
Changed occupation	77 (13.9)	1.81	1.47–2.22	< 0.001	1.82	1.48–2.24	< 0.001¶
Wealth index‖							
Poor	177 (31.6)	Ref	1	–	–	–	–
Middle	223 (39.9)	0.97	0.81–1.15	0.698	1.05	0.88–1.24	0.600
Rich	159 (28.4)	0.98	0.81–1.19	0.917	1.01	0.84–1.23	0.841
COVID-19 testing availability							
No	335 (59.8)	Ref	1	–	–	1	–
Yes	225 (40.2)	1.10	0.94–1.27	0.216	1.11	0.95–1.31	0.178

aPR, adjusted prevalence ratio; CPR, crude prevalence ratio.

* Prevalence ratios were calculated using modified Poisson regression. The models were adjusted for age, gender, and all variables shown in the table.

† Values are either mean (SD) or frequency (percent).

‡ Participants were farmers, students, not employed, and self-employed.

§ Fifty-six observations missing for Dodoma, Tanzania.

‖ Eight observations (one in Ibadan, four in Lagos, and three in Dar es Salaam) are missing.

¶ Significant at 5% significance level.

### Factors associated with reduced access to health services.

Compared with the urban site in Burkina Faso (Ouagadougou), adults in the urban site of Ethiopia (Addis Ababa) and in rural Ghana (Kintampo) were 58% (aPR 0.42; 95% CI: 0.25–0.70) and 58% (aPR 0.42; 95% CI: 0.26–0.69) less likely to report reduced access to health services due to COVID-19. Those in the rural site in Nigeria (Ibadan) were 66% (aPR 1.66; 95% CI: 1.12–2.28) more likely to report reduced access to health services. A 1-unit increase in the number of under 5 years children in a household was associated with a 23% (aPR 1.23; 95% CI: 1.14–1.33) increased prevalence of reporting interrupted access to healthcare. Adults with tertiary and higher educational status and those who any reported psychological distress were 2.08 times as likely (aPR 2.08; 95% CI: 1.29–3.34) and 1.83 times as likely (aPR 1.83; 95% CI: 1.48–2.27) to report difficulty in accessing healthcare compared with those with no education and those without any psychological distress, respectively. Finally, participants used in wage employment were 33% (aPR 0.67; 95% CI 0.49–0.90) less likely to report interrupted healthcare access due to COVID-19 compared with those not used in wage employment (students, farmers, self-used, and not used) (Table 5).

**Table 5:** Factors associated with reduced access to healthcare services due to the COVID-19 pandemic in five sub-Saharan African countries among 1,396 adults, 2021*

	Reduced access (%)†	CPR	95% CI	*P* value	aPR	95% CI	*P* value
Site							
Ouagadougou, Burkina Faso	52 (20.6)	Ref	1	–	–	1	–
Addis Ababa, Ethiopia	23 (9.1)	0.49	0.31–0.78	0.002	0.42	0.25–0.70	0.001‖
Ibadan, Nigeria	106 (42.1)	1.84	1.38–2.47	0.000	1.66	1.12–2.28	0.010‖
Lagos, Nigeria	50 (19.8)	1.04	0.73–1.47	0.840	0.87	0.56–1.34	0.527
Kintampo, Ghana	21 (8.3)	0.49	0.30–0.79	0.003	0.42	0.26–0.69	0.001‖
Age							
20–29	40 (15.87)	Ref	1	–	–	–	–
30–39	71 (28.17)	1.13	0.79–1.62	0.489	1.14	0.80–1.61	0.475
40–49	92 (36.51)	1.46	1.04–2.04	0.028	1.32	0.94–1.86	0.109
≥ 50	49 (19.44)	0.96	0.65–1.42	0.850	1.00	0.69–1.46	0.996
No. of children < 5 years in household (mean/median/*n*)	0.98/1.1/252	1.15	1.05–1.25	0.002	1.23	1.14–1.33	< 0.001‖
Education							
None	32 (12.7)	Ref	1	–	Ref	1	–
Primary	40 (15.9)	1.13	0.74–1.71	0.566	1.69	1.11–2.57	0.014‖
Secondary	57 (22.6)	1.61	1.09–2.38	0.015	1.79	1.16–2.77	0.009‖
Tertiary and higher	123 (48.8)	2.27	1.61–3.21	< 0.001	2.08	1.29–3.34	0.003‖
Wealth index							
Poor	63 (25.0)	Ref	1	–	–	–	–
Middle	118 (46.8)	1.25	0.94–1.66	0.111	1.26	0.95–1.66	0.103
Rich	71 (28.2)	1.22	0.89–1.66	0.208	1.03	0.75–1.40	0.855
Availability of COVID-19 testing							
No	117 (46.4)	Ref	1	–	–	1	–
Yes	135 (53.6)	1.17	0.94–1.47	0.165	1.24	0.98–1.57	0.075
Wage employment							
No‡	205 (81.3)	Ref	–	–	–	1	–
Yes	47 (18.7)	0.81	0.61–1.05	0.157	0.67	0.49–0.90	0.009‖
Any psychological distress							
No	153 (60.7)	Ref	1	–	–	1	–
Yes	99 (39.3)	1.81	1.45–2.26	< 0.001	1.83	1.48–2.27	< 0.001‖

aPR = adjusted prevalence ratio; CPR = crude prevalence ratio.

* Prevalence ratios were calculated using modified Poisson regression; the models were adjusted for age, gender, and all variables shown in the table (*N* = 1,396); participants in Nouna, Kersa, Dar es Salaam, and Dodoma were excluded due to the small proportion of participants reporting the outcome (< 10%).

† Values are either mean/median/*n* or frequency (%).

‡ Participants were farmers, students, not employed, and self-employed.

‖ Significant at 5% significance level.

## DISCUSSION

In this round 2 survey conducted during the COVID-19 pandemic in July–December 2021, an estimated one-ninth of adults reported difficulty accessing health services and one-fifth reported any psychological distress, with differences among sites across five sub-Saharan African countries. These results suggest that health systems and community psychological distress are less affected by the COVID-19 pandemic compared with the baseline survey conducted in 2020, which could be due to health system adaptations over time or changes in the severity of the pandemic. However, COVID-19 continues to pose a considerable challenge in access to essential healthcare services and psychological distress among communities in SSA.

This study also highlights room for improvement in COVID-19 perceptions and preventive measures across five countries in SSA. Compared with the baseline survey conducted in Burkina Faso, Ethiopia, and Nigeria in 2020, in this survey participants reported practicing fewer unscientific methods of preventing COVID-19, such as steaming or using saunas, drinking lemon tea and consuming ginger, and taking vitamins. The proportion of participants reporting using certain evidence-based preventive measures such as canceling or changing travel plans, regular handwashing, and social distancing also decreased. At the same time, self-reported compliance in wearing masks increased in Burkina Faso, Ethiopia, and Nigeria in this survey compared with the baseline. These trends may reflect changes in perceptions and behaviors as new waves of COVID-19 infections surged and fell across the survey rounds.^7^^,^^39^ These findings align with other studies in Ethiopia and Nigeria, showing a decline in and lack of interest in using COVID-19 preventive measures.^22^^,^^40^^,^^41^ These findings also highlight the need for continuous efforts to disseminate accurate health information and education to the community to mitigate the pandemic.^42^^,^^43^

In this survey, we found that the overall proportion of any psychological and mild psychological distress was lower than in the baseline survey (20% and 15.6%, respectively, in round 2, compared with 26% and 21% in round 1). However, moderate and severe psychological distress levels were comparable and remained consistently low. This trend did not apply to Ibadan and Lagos, where there was a slight increase in severe forms of psychological distress outcomes (6% and 10%, respectively, in round 2 compared with 2.9% and 3.1% in round 1).^16^

We found lower levels of psychological distress than in other studies during the pandemic in low- and middle-income countries.^44^^,^^45^ For example, other studies found a 30% prevalence of mild psychological stress, 7% prevalence of anxiety, and 5% prevalence of depression during the COVID-19 pandemic in the general community, using the Depression Anxiety and Stress scale, Generalized Anxiety Disorder–7, and Patient Health Questionnaire–9 measures. A nationwide survey in Iran found higher levels of anxiety (20%) and depression (15%) compared with the levels in our study (6.4% for anxiety and 4.8% for depression).^46^ Differences between our results and other studies could be related to the severity of the pandemic in different locations and at different time points, the extent of continued economic impacts, and the level of stringency of lockdowns and prevention measures at the time of the study.^47^^–^^49^ For example, on December 27, 2021, Ethiopia had the highest stringency index of 40.7 (range from 0 to 100, with 100 = strictest prevention measures) compared with 43.2, 38.0, 13.9, and 8.3 for Ghana, Nigeria, Burkina Faso, and Tanzania, respectively.^47^

Participants from Ouagadougou and Kintampo were more likely to experience any psychological distress compared with the reference category of those in Nouna. These findings could be related to differences across sites in health systems, COVID-19 caseloads, and the financial toll and stress of the pandemic in these different settings.^18^^,^^50^ Additionally, adults engaged in wage employment were less likely to experience any psychological distress than their counterparts (farmers, students, self-employed, and not employed). This could be because those with job stability and a stable income may be more resilient and less vulnerable to the economic shocks created by the pandemic.^6^^,^^51^^,^^52^ We also found that family size was positively associated with psychological distress, which agrees with other studies that reported the number of household members as a risk of psychological distress.^46^^,^^53^^–^^56^ As family size increases, it could be that the stressors and challenges for fulfilling the needs of the family also increase.^54^ Although the pandemic’s impact on psychological distress may be decreasing, tailored public health action is needed during and beyond the pandemic to improve psychological well-being and mental health because there are huge disparities across countries, and a considerable proportion of the community is still suffering from psychological distress.^57^^,^^58^

We found that about one in nine participants reported difficulty accessing at least one type of essential healthcare service due to COVID-19 during our survey (July–December 2021). Compared with other essential health services, children’s immunization services were reported to be difficult to access by the highest proportion of participants. In our previous survey in 2020, one in four adults in Burkina Faso, Ethiopia, and Nigeria reported reduced access to care.^26^ Other studies also found a comparable proportion of difficulty accessing healthcare in that timeframe.^29^^,^^59^^,^^60^ From comparing surveys in 2020 and 2021, it appears that interruptions to healthcare due to COVID-19 may have decreased.^26^ Our finding is also in line with the WHO second round global pulse survey that reported a reduction in the magnitude and extent of healthcare disruption in 2021 compared with 2020.^61^ This reduction may be due to implementation of mitigation strategies for continuing essential health services, easing of lockdowns and preventive measures as the pandemic progressed, the emergence of multiple COVID-19 vaccines, and the variations in COVID-19 caseloads in different parts of SSA.^43^^,^^62^^–^^65^ In addition, very small proportions of participants reported difficulty in accessing healthcare in urban and rural areas of Tanzania (Dodoma and Dar es Salaam), which is consistent with evidence that the stringency of prevention measures was very low in Tanzania at the time of the study and caseloads were decreasing.^47^

We found several factors that were significantly related to self-reported difficulty in access to health services. The number of children aged under 5 years in a household was associated with an increased likelihood of reporting interruptions in healthcare access due to COVID-19. Adults with higher educational status and those in wage employment were also more likely to report difficulty in healthcare access, which could be due to higher health-seeking behaviors of higher income and more educated adults.^28^^,^^66^

We also found that only one-third of participants reported having access to COVID-19 testing, and 16.3% reported ever testing for COVID-19. Additionally, we found a significant disparity in COVID-19 testing availability between rural and urban sites in Burkina Faso, Ethiopia, and Tanzania. For example, more than half of the participants (54%) in Nouna and most of the participants in Addis Ababa (85%) reportedly had access to COVID-19 testing compared with 25% in Ouagadougou and 4% in Kersa. These disparities in testing could lead to underreporting and undetected community transmission of the SARS-CoV2 virus.^67^^,^^68^ Some challenges in scaling the testing and diagnostic facilities for COVID-19 in SSA include gaps in service delivery and health systems, unskilled staffing, poor healthcare financing, sociocultural values, and the absence of infrastructure and technology to initiate and maintain diagnostic services.^69^^–^^71^ This finding indicates the need for reform programs for strengthening and sustaining healthcare systems through appropriate health policy, adequate governmental focus and investment, and capacity-building in human resources, governance, and financing.

A limitation of this study is that we do not have baseline data in this population on healthcare utilization and access before COVID-19; therefore, we can only compare this study to the 2020 study conducted during the COVID-19 pandemic (round 1). In addition, all data collected were self-reported and subject to nonresponse, recall, and social desirability biases. The study population was not selected to be representative of the overall study communities and countries; however, we included diverse urban and rural sites from five countries to increase the generalizability of the findings. In addition, the cross-sectional design of our survey limits our ability to make causal inferences about the effects of the COVID-19 pandemic on psychological distress, healthcare access, and COVID-19 prevention measures; however, reporting these findings is still valuable to help policymakers track these measures to prioritize areas for intervention. Due to limits in the duration of the phone survey, we were not able to assess all types of health services and all factors that may influence psychological distress and access to healthcare.

A strength of this study is the use of CATI surveys to enable remote and rapid data collection during the COVID-19 pandemic.^30^ CATI is the gold standard method for telephone interviewing and has produced results similar to phone-based surveys.^30^ In addition, including participants from urban and rural areas and five sub-Saharan African countries and using standardized tools and methods across sites allowed us to assess and compare psychological distress, COVID-19 preventive measures, and healthcare access across nine diverse settings. The findings of this study will be helpful for healthcare managers and officials to build resilient healthcare systems and inform future research monitoring and mitigating the negative impacts of pandemics across the African continent.

In conclusion, this study suggests that despite some improvements as the COVID-19 pandemic has progressed, psychological distress challenges and interruptions in access to essential health services persist in five sub-Saharan African countries. The extent of these impacts dramatically differs in each country studied, and targeted action is needed to restore full access to health services and improve psychological distress. The low accessibility of COVID-19 testing and the low uptake of testing in SSA are other areas that need urgent attention. In addition to mitigating COVID-19 impacts on psychological distress and healthcare access, efforts to increase COVID-19 vaccination rates and compliance with preventive measures may help decrease the pandemic’s long-term impact and ease the health and economic burden of COVID-19 in SSA.

## Supplemental files


Supplemental materials
