# Supplementary figures and images for: COVID-19 Preventive Practices, Psychological Distress, and Reported Barriers to Healthcare Access during the Pandemic among Adult Community Members in Sub-Saharan Africa: A Phone Survey
Source: Am J Trop Med Hyg. 2022 Dec 12;108(1):124–36. doi: 10.4269/ajtmh.22-0349 (PMC9833061; doi:10.4269/ajtmh.22-0349)

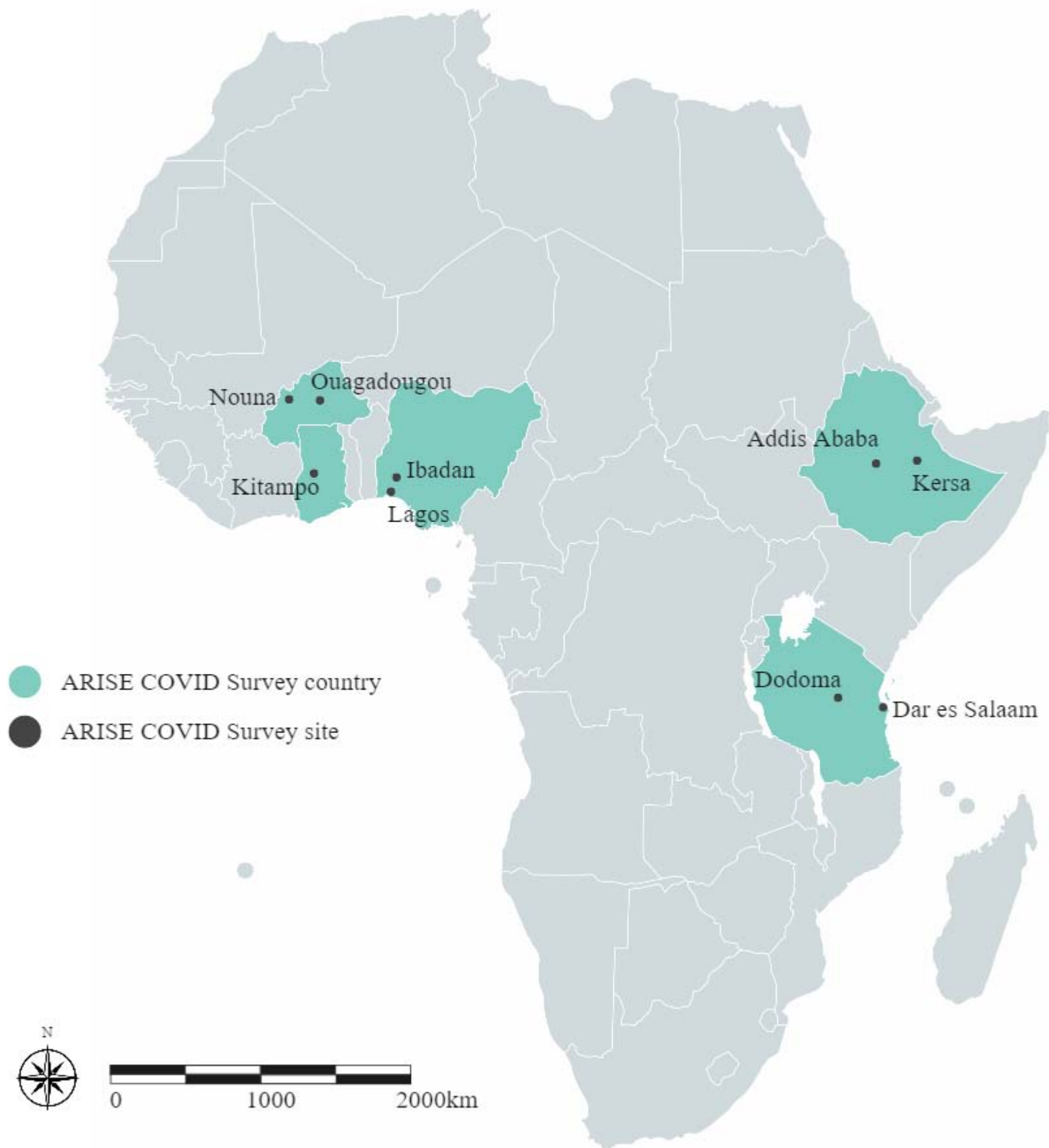

Supplement: Supplementary file 1 [file tpmd220349.SD1.pdf]
